# Aging Modulates the Hemispheric Specialization during Word Production

**DOI:** 10.3389/fnagi.2017.00125

**Published:** 2017-05-09

**Authors:** Elena Hoyau, Naila Boudiaf, Emilie Cousin, Cedric Pichat, Nathalie Fournet, Alexandre Krainik, Assia Jaillard, Monica Baciu

**Affiliations:** ^1^CNRS LPNC UMR 5105, Laboratoire de Psychologie et Neurocognition, Université Grenoble AlpesGrenoble, France; ^2^UMS IRMaGe CHU, Université Grenoble AlpesGrenoble, France; ^3^LPNC UMR 5105, Université Savoie Mont BlancChambéry, France; ^4^Grenoble Institute of Neuroscience, Université Grenoble AlpesGrenoble, France

**Keywords:** object naming, aging, fMRI, hemispheric specialization, LAPA, HAROLD

## Abstract

Although older adults exhibit normal accuracy in performing word retrieval and generation (lexical production; e.g., object naming), they are generally slower in responding than younger adults. To maintain accuracy, older adults recruit compensatory mechanisms and strategies. We focused on two such possible compensatory mechanisms, one semantic and one executive. These mechanisms are reflected at inter- and intra-hemispheric levels by various patterns of reorganization of lexical production cerebral networks. Hemispheric reorganization (HR) changes were also evaluated in relation to increase naming latencies. Using functional magnetic resonance imaging (fMRI), we examined 27 healthy participants (from 30 years to 85 years) during an object naming task, exploring and identifying task-related patterns of cerebral reorganization. We report two main results. First, we observed a left intra-hemispheric pattern of reorganization, the left anterior-posterior aging (LAPA) effect, consisting of supplementary activation of left posterior (temporo-parietal) regions in older adults and asymmetric activation along the left fronto-temporal axis. This pattern suggests that older adults recruit posterior semantic regions to perform object naming. The second finding consisted of bilateral recruitment of frontal regions to maintain appropriate response times, especially in older adults who were faster performers. This pattern is discussed in terms of compensatory mechanism. We suggest that aging is associated with multiple, co-existing compensation and reorganization mechanisms and patterns associated with lexical production.

## Introduction

Anomia and tip-of-the-tongue phenomena are frequently reported by older adults in daily life (Perlmutter, 1978; Zelinski et al., 1980; Cavanaugh et al., 1983). They reflect difficulty in retrieving and generating words (Obler and Albert, 1980; Burke and Shafto, 2004; Salthouse and Mandell, 2013). However, despite these frequent subjective complaints, the objective evidence for lexical production and naming deficits in aging varies according to the task and material (Ska and Goulet, 1989; Goulet et al., 1994; Obler et al., 2010; Baciu et al., 2016; Votruba et al., 2016). For instance, object naming is preserved longer in aging, compared to naming famous people (Cohen and Faulkner, 1986; Evrard, 2002; James, 2004). Boudiaf et al. (2016) have observed that older adults name objects as accurately as younger ones, despite longer naming latencies. These results indicate possible dissociation between accuracy (i.e., number of correct responses) and latencies (i.e., time required to produce the appropriate word). At the cerebral level, aging modulates cerebral activity (Greenwood, 2007), reflected either by increased or decreased activity, or by additional recruitment of regions that are not usually engaged by similar tasks in younger adults (e.g., Cabeza, 2002; Greenwood, 2007; Park and Reuter-Lorenz, 2009; Lövdén et al., 2010; Grady, 2012). These modifications are reflected in multiple patterns of inter- and intra-hemispheric cerebral reorganization (e.g., Grady et al., 1994; Cabeza, 2002; Davis et al., 2008). In our study, we sought to determine whether the dissociation of naming performance in aging (i.e., preservation of naming accuracy despite longer latencies) is associated with intra- and inter-hemispheric cerebral reorganization mechanisms.

Two hypotheses may explain why older adults may show only limited decline in object naming accuracy: one semantic and the other executive. The semantic hypothesis is based on the fact that the experience that older people have acquired over the years, resulting in a larger vocabulary and semantic memory (Verhaeghen, 2003), is associated with an increased number of shared features and representations in semantic memory (Laver and Burke, 1993). Therefore, the relative preservation of object naming in older adults might be explained by a supplementary contribution of semantic processes that facilitate lexico-semantic retrieval during naming (Boudiaf et al., 2016). In line with the semantic hypothesis, using functional magnetic resonance imaging (fMRI), Baciu et al. (2016) reported higher supplementary bilateral posterior temporo-parietal activity for object naming in older adults compared to younger adults, suggesting greater involvement of lexico-semantic processes. This is in agreement with findings from Ansado et al. (2013) for lexical production, and Lacombe et al. (2015) for language comprehension. In addition, Lacombe et al. (2015) reported that increased posterior activity observed in older adults reflects age-related reorganization of networks responsible for the conceptual retrieval and semantic representations of words. Ansado et al. (2013) suggested that preserved lexical production in older adults could be explained by greater support from semantic processes in temporal regions (Verhaeghen, 2003; Patterson et al., 2007). In terms of inter- and intra-hemispheric reorganization (HR), the semantic hypothesis predicts increased temporo-parietal and decreased frontal activity. This HR mechanism can be put in perspective with the posterior-anterior shift in aging (PASA, Davis et al., 2008) that posits that older adults exhibit greater anterior frontal and decreased posterior occipital activation, reflecting executive-based compensation (frontal regions) in the context of a sensory deficit (occipital regions; Grady et al., 1994; Park et al., 2004; Davis et al., 2008). Other PASA variants reported decreased activity in regions other than the occipital lobe, such as medial temporal areas (Gutchess et al., 2005; see Morcom and Johnson, 2015). According to Ansado et al. (2013), age-related increases in temporo-parietal or frontal activity might depend on the particular cognitive processes involved. Along the same lines, the second hypothesis (i.e., executive hypothesis) posits a supplementary recruitment of executive functions in older adults to perform the task (Wingfield and Grossman, 2006; Helder et al., 2016). Using fMRI, Wierenga et al. (2008) revealed supplementary right hemispheric activity in older adults, variably correlated with the performance. Right pre-central activity was negatively correlated, whereas right inferior frontal activity was positively correlated with task accuracy. This reduced inter-hemispheric frontal asymmetry in aging was interpreted as a difficulty in retrieving words, resulting from a decline in executive function (Wierenga et al., 2008). In terms of inter- and intra-hemispheric HR, increased frontal activity was related to hemispheric asymmetry reduction in older adults (HAROLD; Cabeza, 2002) model. According to the HAROLD model, compared to younger adults, older adults exhibit a lower degree of hemispheric asymmetry, mainly in prefrontal regions (Cabeza, 2002; Rajah and D’Esposito, 2005; Park and Reuter-Lorenz, 2009). According to HAROLD, the supplementary involvement of right prefrontal regions reflects engagement of compensatory executive mechanisms to maintain performance. Furthermore, the HAROLD model suggests a dissociation between high- and low-performing older adults, with the latter showing less hemispheric asymmetry reduction than the former (Cabeza et al., 2002).

For naming latencies, Obler et al. (2010) observed that object naming latencies related to aging correlated to white matter density in both frontal and temporo-parietal regions using voxel-based morphometry (VBM). This suggests that the anatomical basis of naming processes with aging is organized along fronto-temporo-parietal or antero-posterior axes. Other authors have shown that the left antero-posterior axis was related to task demands (Wierenga et al., 2008; Galdo-Alvarez et al., 2009; Diaz et al., 2016). By comparing comprehension (low task-demand) vs. production (high task-demand) tasks, Diaz et al. (2016) observed that the higher the task demands, the stronger the older adults recruit frontal regions. This result suggests that older adults increase the recruitment of executive functioning to maintain lexical production, as during naming. However, older adult’s performance is highly variable (Hultsch et al., 2002). Whereas some older adults show decreased performance, others tend to show similar performance, as compared to younger adults. Such variability may be explained in terms of cognitive reserve (i.e., higher educational level or more flexible neural networks; Stern, 2002, 2009), allowing older adults to preserve high cognitive functioning. Indeed, Cabeza et al. (2002) have observed that in high-performing older adults, a memory task recruited both left and right frontal regions, whereas low-performing older adults recruited only left frontal regions. Thus, the aforementioned HAROLD pattern may be related to higher cognitive reserve, suggesting that differential frontal recruitment, related to executive functioning, can be expected in older adults. Wierenga et al. (2008) showed that in older adults correlations between BOLD-contrast signals and response latency were found in several frontal regions, suggesting supplementary selection of lexical representations by older adults. However, the distribution of latencies as a function of the BOLD-contrast response (see Wierenga et al., 2008) appeared to be driven by extreme values, weakening the statistical validity of their results. In this study, we evaluated the influence of age on naming latencies (i.e., between young and old adults) and cognitive reserve (i.e., within older adults only). The effect of cognitive reserve on naming latencies in older adults was explored by dividing the older group into two groups, slower (longer latencies or response times) or faster (shorter latencies) adults.

In summary, older adults show preserved naming accuracy but longer naming latencies compared to younger adults, suggesting a dissociation of neural substrates for accuracy and latencies. Our main goal was to determine HR mechanisms that may account for this dissociation, with two objectives. To explore the respective role of semantic and executive mechanisms in naming accuracy preservation with aging (Objective 1), we inferred these mechanisms *using* HR indices, with semantic mechanisms reflected by greater posterior (temporo-parietal) asymmetry, and executive mechanisms reflected by greater anterior (frontal) asymmetry. To determine which HR mechanisms are related to naming latencies (Objective 2), we compared the effect of naming latencies between younger and older adults, and between faster and slower older adults.

## Materials and Methods

### Participants

Among the 30 participants initially recruited, 27 (8 females) aged from 30 to 85 (*M* = 56.22 years old, SD = 17.53, variation coefficient = 0.31) were finally retained, divided into two groups: the Younger Group (YG; *n* = 13, *M* = 40.07 years old, SD = 8.33, 5 females) and the Older Group (OG; *n* = 14, *M* = 71.21 years old, SD = 6.93, 3 females). All participants were right-handed (Edinburgh Handedness Inventory; Oldfield, 1971) and had normal or corrected-to-normal vision. Participants were cognitively unimpaired (Mini Mental State Examination, MMSE; Folstein et al., 1975), had no psychiatric symptoms (Hospital Anxiety and Depression, HAD; Zigmond and Snaith, 1983) or episodic memory deficit (“5 words” test, Dubois et al., 2002). All participants were highly educated (Poitrenaud questionnaire; Kalafat et al., 2003) and were native French speakers. They gave their written informed consent for the study, which was approved by the local ethics committee (CPP no. 2014-A00569-38). Demographic information and inclusion criteria are mentioned in Table 1.

**Table 1:** **Demographic information and inclusion criteria for all participants**.

	YG *N* = 13 5 females		OG *N* = 14 3 females		Mann-Whitney tests	
	Mean	*SD*	Mean	*SD*	*Z*	*p*
Age	40.07	*8.33*	71.21	*6.93*	−4.418	**<0.01**
Socio-cultural	4.00	*0.00*	3.85	*0.36*	−1.390	0.550
Laterality	85.00	*16.99*	90.00	*11.87*	−0.817	0.430
MMSE	29.53	*0.66*	29.07	*1.20*	−1.024	0.375
HAD_A	5.92	*2.46*	6.21	*1.96*	−0.098	0.943
HAD_D	2.46	*1.56*	3.92	*2.84*	−1.329	0.202
Dubois	10.00	*0.00*	9.92	*0.26*	−0.964	0.756

*Mann-Whitney tests were performed between younger and older participants. Analyses show that younger and older participants differ only on age. Significant results are in bold. Abbreviation: YG, Younger group; OG, Older group; MMSE, Mini-Mental State Evaluation; HAD_A, Hospital Anxiety and Depression scale_Anxiety; HAD_D, Hospital Anxiety and Depression scale_Depression*.

### Cognitive Assessment

Several neuropsychological tests were administered to evaluate cognitive domains, including language, memory, visual processing, and executive function, that are commonly used to measure cognitive function associated with naming (Duffau et al., 2014). Language was assessed using verbal fluency (generation of words that are related to the same semantic category; Cardebat et al., 1990), the verbal automatisms test (completion of overlearned French expressions by the participants; Beauregard, 1971), and Mill-Hill B vocabulary scale (explanation of words meaning and selection of the appropriate synonym for a word among a list; Deltour, 1993). Memory was assessed using forward and backward digit span tests (short-term and working memory; Wechsler, 1997) and the MacNair questionnaire to assess subjective memory complaints (McNair and Kahn, 1983). Visual processing was evaluated using the version A of the Trail Making Test (TMT-A; Tombaugh, 2004). Finally, executive functioning was assessed using Version B of the TMT (TMT-B; Tombaugh, 2004). We computed a difference score between the TMT-B and the TMT-A, referred as the “executive score”, as mentioned in the next sections of the manuscript (Corrigan and Hinkeldey, 1987). This executive score reflected cognitive flexibility, and removed simple sequencing, visual scanning and psychomotor functioning effects.

### Object Naming: Stimuli and Procedure

**Figure 1:**
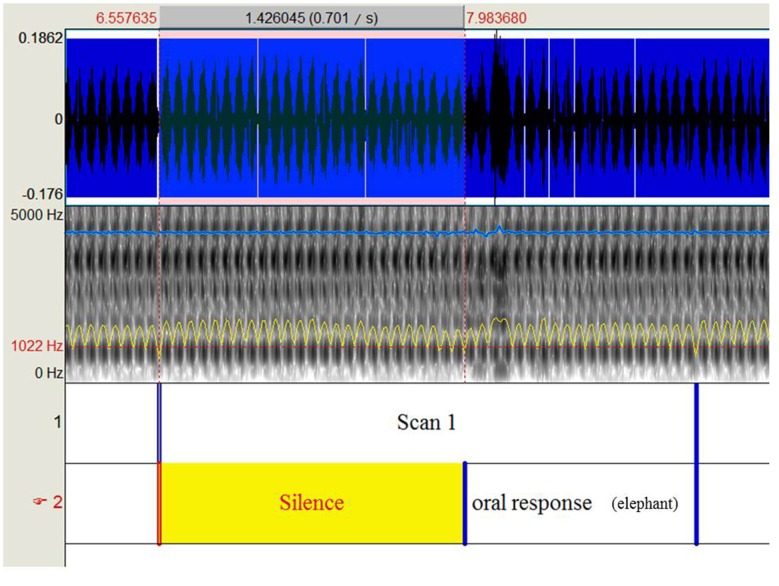
**Shows the procedure to extract response times recorded during the object naming task under functional magnetic resonance imaging (fMRI)**.

The participants performed an object naming task with 80 black and white drawings of objects and animals (DO-80 test; Metz-Lutz et al., 1991) using a blocked design alternating four task and control periods. Twenty images lasting 2 s each with an inter-stimulus interval of 500 ms were presented during each task period, and participants were instructed to name the images as accurately and rapidly as possible. During each control period, 20 simple images (circles and squares) were presented and participants were instructed to respond either “square” or “round.” Stimuli were displayed using E-prime (E-prime Psychology Software Tools Inc., Pittsburgh, PA, USA) and were projected onto a screen behind the magnet using a video projector. Oral responses were recorded via an MRI-compatible microphone (FORMI™ II, version 1.2) attached to the coil. We used Praat software (Boersma and Weenink, 2001) to measure the response times for each item and each participant. Specifically, we delimited 160 periods of 2.5 s from each audio file (one per participant) that correspond to the repetition time for scan acquisition. Within each identified scan, we delimited the beginning of oral response according to the audio-spectrogram (Figure 1). Response latencies were calculated based on the difference between the beginning of oral response and the beginning of the scan, given that pictures were presented at the beginning of each scan. Based on oral responses, the correct response rate (% of CR) and the mean reaction time (RT, in milliseconds) for correct responses were calculated. The run lasted 7.06 min.

### Functional MRI Acquisition

The study was performed in a whole-body 3T MR scanner (Philips Achieva) with a 32-channel head coil. For functional scans, the manufacturer-provided gradient-echo/T2*-weighted EPI method was used. Forty-four interleaved axial slices parallel to the bi-commissural plane were acquired. Slice thickness was 3 mm. The in-plane voxel size was 2.3 × 2.3 mm (220 × 220 mm field of view acquired with 88 × 85 pixel data matrix, reconstructed with zero filling to 96 × 96 pixels). The main sequence parameters were TR = 2.5 s, TE = 30 ms, and flip angle = 80°. A T1-weighted high-resolution three dimensional anatomical volume was acquired, via a 3D T1 TFE sequence (field of view = 256 × 240 × 160 mm; resolution: 0.89 × 0.89 × 1 mm; acquisition matrix: 272 × 250 × 160 pixels; reconstruction matrix: 288 × 288 × 160 pixels).

### Data Processing

#### Cognitive Scores and Naming Performance

Cognitive values were within normal neuropsychological ranges for all but one participant (MMSE < 24), who was then excluded from the study. Two other participants were also excluded owing to abnormal behavioral responses (RTs > 2 SD). Twenty-seven participants were thus retained. For recall, we aimed to determine: (1) which strategy was used by older adults to maintain equivalent naming accuracy than younger adults (semantic or executive); and (2) which HR mechanisms might explain longer naming latencies: (a) in older adults compared to younger adults; and (b) in slower older adults compared to faster older adults (in relation to cognitive reserve). For (1), we inferred the two strategies, semantic vs. executive, from HR indices reflecting patterns of HR. For their calculation, see below. The cut-off between YG and OG was based on the median age of 59 years. To test the effect of naming latencies, OG was divided into two groups based on the naming latencies, with a faster group (OG/RT+; *n* = 7, *M* = 830.52 ms, SD = 53.02) and a slower group (OG/RT−; *n* = 7, *M* = 1004.61 ms, SD = 68.57). The OG/RT+ and OG/RT− did not differ in terms of age (*Z* = −0.384, *p* = 0.710) and other cognitive scores (see Table 2), except for naming latencies (*Z* = −3.13, *p* = 0.001).

**Table 2:** **Non-parametrical analyses between faster older adults (OG/RT+) and slower older adults (OG/RT−) on the object naming task on inclusion criteria, cognitive scores and object naming performance**.

		OG/RT+ *N* = 7		OG/RT− *N* = 7		Mann-Whitney test	
		Mean	*SD*	Mean	*SD*	*Z*	*p*
	Age	70.71	9.14	71.71	4.46	−0.384	0.710
Inclusion criteria	Socio-cultural	3.85	*0.37*	3.85	*0.37*	0.00	1.00
	Laterality	90.42	*9.88*	89.57	*14.39*	−0.332	0.805
	MMSE	28.71	*1.60*	29.42	*0.53*	−0.556	0.620
	HAD_A	5.71	*1.70*	6.71	*2.21*	−1.110	0.318
	HAD_D	3.42	*2.82*	4.42	*2.99*	−0.642	0.535
	Dubois	9.85	*0.37*	10.00	*0.00*	−1.00	0.710
Naming	**RTs**	**830.52**	**53.02**	**1004.61**	**68.57**	**−3.130**	**0.001**
	%CR	98.86	1.46	98.71	1.38	−0.335	0.805
Perceptuo-motor	TMT-A	42.00	14.25	52.28	7.56	−1.599	0.128
Executive	[TMT-B] minus [TMT-A]	57.00	25.31	52.00	*11.71*	−0.576	0.620
	Verbal fluency	21.28	*8.03*	19.00	*5.50*	−0.388	0.710
Language	Verbal automatisms	35.14	*2.03*	34.42	*1.98*	−0.581	0.620
	Mill-Hill	40.00	*2.51*	10.14	*1.67*	−0.258	0.805
Memory	Forward digit span	8.71	*2.21*	8.71	*2.28*	0.000	1.000
	Backward digit span	8.57	*3.45*	9.00	*2.08*	−1.121	0.318
	MacNair	13.85	*5.04*	15.85	*6.89*	−0.563	0.620

*Analyses show that the two groups differ only on RTs for naming (in bold). Abbreviation: OG, Older group; MMSE, Mini-Mental State Evaluation; HAD_A, Hospital Anxiety and Depression scale_Anxiety; HAD_D, Hospital Anxiety and Depression scale_Depression; TMT-A, Trail Making Test, version A; TMT-B, Trail Making Test, version B*.

##### Objective 1: naming accuracy

YG and OG were compared on cognitive scores (i.e., language, memory, executive, and perceptuo-motor processes) and naming performance (RTs, %CR) using Mann-Whitney tests. We also performed Mann-Whitney tests on the HR indices between the YG and the OG, to determine which strategy was used to maintain naming accuracy.

##### Objective 2: naming latencies

Difference in HR indices between YG and OG (based on Objective 1) were controlled for by including RT as a covariate during naming, determining whether the age difference was explained by naming latencies. We also performed Mann-Whitney tests on HR indices between OG/RT+ and OG/RT− to evaluate the cognitive reserve mechanism at intra- and inter-hemispheric levels.

#### Neuroimaging

##### Cerebral activation during object naming

###### Pre-processing

Preprocessing was performed using SPM12 (Welcome Department of Imaging Neuroscience, London, UK^1^) implemented in MATLAB 2014 (Mathworks Inc., Sherborn, MA, USA). As the use of a study-specific template (SST) is recommended for aging studies to avoid methodological bias attributable to morphometric inter-individual differences (Huang et al., 2010; Fillmore et al., 2015), we first created an SST. The T1-weighted anatomical volumes were coregistered to the mean image created by the realignment of the functional images and segmented via DARTEL using the six tissue probability maps. We generated an SST by matching all of the tissue class images provided during the segmentation step, and then normalized this template to the MNI space. The T1-weighted anatomical volumes were normalized to the SST. Then temporal correction of the realigned functional images was performed. The deformation field generated by the segmentation was subsequently used for the normalization of functional volumes. Finally, each functional volume was smoothed by means of an 8-mm full width at half maximum (FWHM) Gaussian kernel.

###### Statistical first-level analyses

The fMRI signal was analyzed using the general linear model at an individual level (Friston et al., 1994, 1995). For each participant, two conditions of interest (task, control) were modeled as two regressors, constructed as box-car functions convolved with a canonical hemodynamic response. Movement parameters derived from realignment correction were entered in the design matrix as six additional regressors of no interest, in order to account for motion-related variability. The time series for each voxel were high-pass filtered (1/128 Hz cutoff).

###### Statistical second-level analyses

One-sample *t*-test group analysis was performed to obtain activation for the main contrast of interest (task > control) for all participants (*N* = 27; *k* = 5; *p* < 0.05 corrected; *t* = 5.9). Activated regions were identified and labeled via the macroscopic parcellation of the MNI single subject reference brain (Tzourio-Mazoyer et al., 2002).

###### ROI analysis

To determine the effect of age and naming latencies on intra and inter-hemispheric asymmetry, we first defined anatomical regions of interest (ROI) based on previously reported results for object naming (Indefrey and Levelt, 2004; Indefrey, 2011) and semantic memory (Binder et al., 2009). We obtained the following ROIs: (a) *frontal level*: inferior frontal gyrus (IFG), middle frontal gyrus (MFG), superior frontal gyrus (SFG), supplementary motor area (SMA); (b) *temporal level*: postero-inferior temporal/ fusiform gyrus (FG), middle-inferior temporal gyrus (ITG), middle temporal gyrus (MTG), anterior temporal lobe (ATL) and hippocampus; (c) *parietal level*: inferior parietal lobule (IPL). We also included the insula, given its role in lexical production (Indefrey and Levelt, 2004). Second, to get ROIs based on naming-task related activity, we defined the ROIs based on the peak activity in our population. To account for anatomo-functional variability of the activation related to age, we defined the coordinates of each ROI separately, for each participant, using the leave-one-out (LOSO) method (Esterman et al., 2010; Prevost et al., 2017). Accordingly, 27 one-sample *T*-tests analyses (Task > Control contrast) were run, each one leaving out one participant. Based on these analyses, we determined the peak of activation of each ROI for the participant left aside. Each ROI was symmetrically defined in the left and right hemispheres. Each ROI was defined as a sphere (6 mm radius; Heath et al., 2012), centered on the activation peak. For each ROI and each participant, we extracted the mean beta values (i.e., BOLD-contrast signal) for the task and the control condition. BOLD-contrast values, calculated from the difference task minus control, were retained for further analyses. These BOLD-contrast differential values were separated into two groups of category regions (CR), an anterior CR (grouping 4 frontal regions and insula) and a posterior CR (grouping 6 temporo-parietal regions). Table 3 shows the mean spatial coordinates and standard deviation for each ROI. Based on BOLD-contrast task-specific values for each right and left region located in either the anterior or the posterior CR, we defined four HR indices, two at an intra-hemispheric level (anterior vs. posterior) and two at an inter-hemispheric level (left vs. right). The intra-hemispheric HR indices were as follows: left anterior-posterior asymmetry index (**L_AP_**), calculated as the difference between left anterior and left posterior CR, and right anterior-posterior asymmetry index (**R**_AP_), calculated as the difference between right anterior and right posterior CR. For an illustrative purpose, we mention below the formula used for calculating the L_AP_ index:

(1) $$L_{\text{AP}}\text{ = }\frac{\sum_{\text{1}}^{\text{5}}left\;anteriror}{\text{5}}-\frac{\sum_{\text{1}}^{\text{6}}left\;posterior}{\text{6}}$$

including left anterior^1^ = left IFG, left anterior^2^ = left MFG, left anterior^3^ = left SFG, left anterior^4^ = left Insula, and left anterior^5^ = left SMA; left posterior^1^ = left FG, left posterior^2^ = left MTG, left posterior^3^ = left ITG, left posterior^4^ = left ATL, left posterior^5^ = left hippocampus, and left posterior^6^ = left IPL. The inter-hemispheric HR indices were as follows: anterior left-right asymmetry index (**A_LR_**), calculated as the difference between left anterior and right anterior CR, and posterior left-right asymmetry index (**P**_LR_), calculated as the difference between left posterior and right posterior CR. Each of these four HR indices was used for correlation analyses with age, naming performance, and cognitive scores.

**Table 3:** **Mean and standard deviation for activation peaks for each region of interest (ROI) labeled according to the AAL atlas and to Brodmann Areas**.

		*x*	*y*	*z*	AAL label	Brodmann Area
Anterior	Left insula	−33.7 (±1.25)	22.0 (±4.34)	9.0 (±2.03)	Insula_L	BA 13
	*Right insula*	33.7 (±1.25)	22.0 (±4.34)	9.0 (±2.03)	Insula_R	BA 13
	Left IFG	−54.9 (0.38)	24 (±0.38)	23.0 (±0)	Frontal_Inf_Tri_L	BA 45
	*Right IFG*	54.9 (±0.38)	24 (±0.38)	23.0 (±0)	Frontal_Inf_Tri_R	BA 45
	Left MFG	−29.8 (±0.96)	10.0 (±0)	32.3 (±1.73)	Frontal_Mid_L	undefined
	*Right MFG*	29.8 (±0.96)	10.0 (±0)	32.3 (±1.73)	Frontal_Inf_Oper_R	undefined
	Left SFG	−11.0 (±0)	20.4 (±1.88)	42.1 (±3.34)	Frontal_Sup_L	BA 32
	Right SFG	14.0 (±0)	12.0 (±0.38)	47.0 (±0)	Frontal_Sup_R	undefined
	Left SMA	−8.7 (±0.64)	18.5 (±1.11)	47.3 (±1.27)	Supp_Motor_Area_L	undefined
	Right SMA	12.2 (±1.87)	13.9 (±0.38)	47.0 (±0)	Supp_Motor_Area_R	undefined
Posterior	Left FG	−46.2 (±0.72)	−55.5 (±3.66)	−15.7 (±0.80)	Temporal_Inf_L	BA 37
	Right FG	45.3 (±2.63)	−55.0 (±1.59)	−16.0 (±0)	Temporal_Inf_R	BA 37
	Left ITG	−50.0 (±0)	−50.0 (±0)	−16.0 (±0)	Temporal_Inf_L	BA 20
	Right ITG	51.0 (±0)	−54.1 (±1.39)	−16.0 (±0)	Temporal_Inf_R	BA 20
	Left MTG	−52.8 (±0, 96)	(±0.57)	−18.8 (±0.57)	Temporal_Mid_R	BA 21
	Left ATL	−32.0 (±0)	5.0 (±0)	−25.0 (±0)	Temporal_Pole_Sup_L	BA 38
	Right ATL	35.0 (±0)	5.0 (±0)	−25.0 (±)	Temporal_Pole_Sup_R	undefined
	Left Hipocampus	−26.8 (±0.53)	−11.0 (0.38)	−22.0 (±)	Hippocampus_L	Hippocampus
	Right Hippocampus	25.0 (±0)	−11.0 (±0)	−22.0 (±0)	Hippocampus_R	Hippocampus
	Left IPL	−49.8 (±0.53)	−29.2 (±0.8)	31.5 (±1.6)	SupraMarginal_L	BA 40
	Right IPL	42.8 (±1.03)	−31.8 (±0.9)	32.0 (±0)	Undefined	BA 40

*Abbreviations: BA, Brodmann Area; IFG, inferior frontal gyrus; MFG, middle frontal gyrus; SFG, superior frontal gyrus; SMA, supplementary motor area; FG, fusiform gyrus; ITG, inferior temporal gyrus; MTG, middle temporal gyrus; ATL, anterior temporal lobe; IPL, inferior parietal lobule*.

## Results

This section is structured in three parts: *General results* (aging effects on cognitive performance, naming performance and cerebral activity); *Objective 1: Naming accuracy*, and *Objective 2: Naming latencies*.

### General Results

#### Cognitive Scores

Results of Man-Whitney analyses performed for cognitive scores in young and old age groups are presented in Table 4.

**Table 4:** **Non-parametrical analyses between younger participants (YG) and older participants (OG) on naming performance and cognitive scores**.

		YG *N* = 13		OG *N* = 14		Mann-Whitney tests	
		Mean	*SD*	Mean	*SD*	*Z*	*p*
Naming	**RTs**	**818.59**	**72.06**	**917.56**	**107.83**	**−2.475**	**0.012**
	%CR	98.31	1.88	98.79	1.36	−0.681	0.519
Perceptuo-motor	**TMT-A**	**30.61**	**9.66**	**47.14**	**12.19**	**−3.108**	**0.001**
Executive	**[TMT-B] minus [TMT-A]**	**23.00**	**8.15**	**54.50**	**19.12**	**−3.891**	**<0.01**
	Verbal fluency	28.53	10.99	20.14	6.72	−1.779	0.076
Language	**Verbal automatisms**	**30.66**	**3.25**	**34.78**	**1.96**	**0.321**	**0.001**
	Mill-Hill	38.23	4.30	40.07	2.05	−0.933	0.375
Memory	Forward digit span	10.08	2.67	8.71	2.16	−1.319	0.212
	Backward digit span	9.38	2.32	8.78	2.75	−1.000	0.350
	**MacNair**	**9.84**	**5.56**	**14.85**	**5.89**	**−2.141**	**0.033**

*Significant differences are in bold. Abbreviation: YG, Younger group; OG, Older group; TMT-A, Trail Making Test, version A; TMT-B, Trail Making Test, version B*.

As illustrated in Table 4, we obtained significant differences between YG and OG on the verbal automatisms test (*Z* = −3.321, *p* = 0.001), MacNair questionnaire (*Z* = −2.141, *p* = 0.033), executive score (*Z* = −3.981, *p* < 0.05), and TMT A scores (*Z* = −3.108, *p* = 0.001). The difference between YG and OG was not significant for verbal fluency (*Z* = −1.779, *p* = 0.076), Mill-Hill scores (*Z* = −0.933, *p* = 0.375), and forward (*Z* = −1.319, *p* = 0.212) and backward (*Z* = −1.000, *p* = 0.350) digit span tests. Overall, OG showed preserved language and memory functioning despite increased subjective memory complaints, with decline of executive functioning and general cognitive slowing.

#### Naming Performance

As illustrated in Table 4, Man-Whitney analyses between YG and OG on RTs for object naming revealed that older adults were significantly slower than younger adults (*Z* = −2.473, *p* = 0.012). Importantly, no difference between YG and OG was observed for naming accuracy (*Z* = 0.681, *p* = 0.519).

#### fMRI Activation during Object Naming

**Figure 2:**
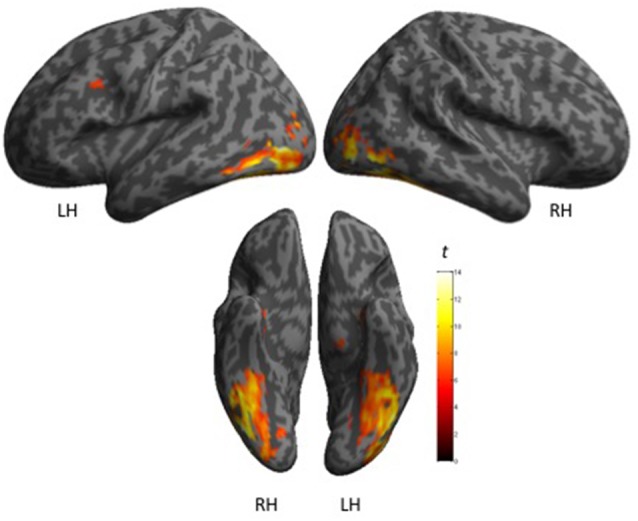
**Shows maps of activation during object naming for all participants (*N* = 27), as projected onto 3D anatomical templates**. The color scale indicates the *t*-value of the activation.

The cerebral network for object naming was obtained by calculating the main contrast, Task vs. Control, that elicited bilateral activation of the FG, ITG and hippocampus, and left activation of the inferior frontal and left pre-central gyri (see Figure 2 and Table 5).

**Table 5:** **Shows main effect of the object naming in all participants (*n* = 27), in terms of peaks of activation for the contrast task > control (one sample *t*-test)**.

Cluster	Label	Peak {mm}			Peak (statistics)	
*k*	AAL	*x*	*y*	*z*	*T*	*Z*
1172	**Occipital_Mid_L**	**−43**	**−80**	**−1**	**14.44**	**7.67**
	*Occipital_Inf_L*	−46	−61	−13	12.67	7.25
	*Fusiform_L*	−41	−66	−13	11.81	7.02
1161	**Occipital_Inf_R**	**39**	**−80**	**−10**	**14.04**	**7.58**
	*Fusiform_R*	37	−71	−13	12.77	7.28
	*Temporal_Inf_R*	44	−57	−16	12.53	7.22
82	**Hippocampus_L**	**−23**	**−6**	**−22**	**8.20**	**5.81**
79	**Hippocampus_R**	**25**	**−9**	**−22**	**7.63**	**5.57**
	*ParaHippocampal_R*	16	−4	−22	7.24	5.39
26	**Precentral_L**	**−39**	**8**	**29**	**7.50**	**5.51**
	*Frontal_Inf_Tri_L*	−41	14	29	7.15	5.35
60	**Temporal_Inf_R**	**−9**	**−27**	**−19**	**7.45**	**5.49**

*The most significant voxels of the cluster are in bold. For each peak, the number of voxels (k), the AAL label, the coordinates (x, y, z; in mm) and the T and Z values are indicated*.

### Objective 1: Naming Accuracy

#### Analyses between HR Indices and Age Groups

As illustrated in Table 6a, our results indicate a significant difference between YG and OG only for the L_AP_ indices (*Z* = −2.329, *p* = 0.019). The difference was not significant for A_LR_ (*p* = 0.128), P_LR_ (*p* = 0.830) and R_AP_ (*p* = 0.239) indices. Compared to the YG, the OG showed greater activity in the left temporo-parietal regions (YG, Mean = −0.21, SD = 0.22; OG, Mean = −0.48, SD = 0.29).

**Table 6:** **Non-parametrical analyses between (a) YG and OG, and (b) OG/RT+ and OG/RT− on hemispheric reorganization (HR) indices**.

(a)		L_AP_	R_AP_	A_LR_	P_LR_
YG *N* = 13	Mean	−0.21	−0.32	0.13	0.02
	SD	0.22	0.32	0.16	0.16
OG *N* = 14	Mean	−0.48	−0.49	0.03	0.02
	SD	0.29	0.32	0.11	0.15
Z		−2.329	−1.213	−1.553	−0.243
*p*		**0.019**	0.239	0.128	0.830
**(b)**		**L_AP_**	**R_AP_**	**A_LR_**	**P_LR_**
OG/RT+ *N* = 7	Mean	−0.54	−0.44	−0.03	0.05
	*SD*	*0.28*	*0.28*	*0.08*	*0.11*
OG/RT− *N* = 7	Mean	−0.43	−0.54	0.10	−0.009
	*SD*	*0.32*	*0.36*	*0.10*	*0.20*
Z		−0.575	−0.319	−2.236	−0.192
*p*		0.620	0.805	**0.026**	0.902

*Significant differences are in bold. For L_AP_ and R_AP_, negative values indicate posterior > anterior asymmetry and positive values indicate anterior > posterior asymmetry. For A_LR_ and P_LR_, negative value indicate right > left asymmetry and positive value indicate left > right asymmetry. Abbreviation: YG, Younger group; OG, Older group; L_AP_, left anterior-posterior asymmetry; R_AP_, right anterior-posterior asymmetry; A_LR_, anterior left-right asymmetry; P_LR_, posterior left-right asymmetry*.

### Objective 2: Naming Latencies

When controlling for naming latencies (ANCOVA analysis), the difference between YG and OG on L_AP_ indices (see above) remained significant (*F*_(1, 26)_ = 6.450, *p* = 0.018). This result indicates that the increase left temporo-parietal asymmetry in OG was not related to increase naming latencies with age.

Mann-Whitney test comparing OG/RT+ and OG/RT− (see Table 6b) indicates that faster older participants (OG/RT+) showed less inter-hemispheric asymmetry on the A_LR_ indices (*Z* = −2.236, *p* = 0.026) than slower older participants (OG/RT−). The difference was not significant for other HR indices (P_LR_, *p* = 0.902; L_AP_, *p* = 0.620; R_AP_, *p* = 0.805).

## Discussion

We aimed to evaluate the effect of normal aging on lexical production, in terms of intra- and inter-HR (HR indices) of cerebral activation during an object naming task. Our main objective was to identify possible compensatory mechanisms that could explain the maintenance of accuracy (%CR, Objective 1) in older participants associated with increased lexical production response latencies (RTs, Objective 2). Our results showed increased recruitment of left posterior temporo-parietal cortex along the left anterior-posterior axis in older, compared to younger, participants. This result is in agreement with the semantic hypothesis, suggesting that older adults might enhance the recruitment of semantic knowledge to maintain naming accuracy. Moreover, in older participants, we found an anterior inter-hemispheric asymmetry that correlated with shorter RTs (i.e., better performance), suggesting that the bilateral anterior frontal regions might reflect compensatory executive-based mechanisms in relation to the cognitive reserve. The following section presents a more detailed and comprehensive discussion of our results, with two parts: (1) aging effects on cognitive performance; and (2) aging effects on HR patterns during object naming in relation to accuracy and response latencies.

### Aging Effects on Cognitive Performance

Based on cognitive scores, our older participants were less efficient for executive functions and visual analysis, in line with other studies showing aging effects for executive functions (Tomer and Levin, 1993; Cepeda et al., 2001; Ashendorf et al., 2008; Turner and Spreng, 2012), perceptual processing and speed of sensory-motor processing (Cerella et al., 1990; Salthouse, 2004). However, in our study, working memory (backward digit span) and verbal short-term memory (forward-digit span) remained intact in older participants, contrary to other findings (Hultsch et al., 1992; Wang et al., 2011; Logie and Morris, 2014). The fact that, in this study, older adults were well educated and had high socio-cultural levels might explain the preservation of some cognitive abilities (Orsini et al., 1986). Despite adequate scores for both short and long term memory, older participants reported subjective memory complaints more frequently. However, it has been shown that subjective memory complaints may be unrelated to objective deficits (Bolla et al., 1991; Mol et al., 2006). Finally, verbal automatisms increased in older participants, suggesting an increase of overlearned knowledge and automatic retrieval processes with age. Age differences for verbal automatisms scores may point to a cohort effect that could potentially skew interpretations of the results (see Froger et al., 2009 who compared verbal automatisms between older groups only, for example). Overall, our findings are in agreement with previous studies suggesting that older adults show a greater decline in executive functions than in language functions (Glisky, 2007; Harada et al., 2013).

### Aging Effects on Hemispheric Reorganization (HR) during Naming in Relation to Accuracy and Response Latency

The main contrast, Task minus Control, revealed that object naming elicited activation of the classical cerebral network reported for this task, including bilateral FG (perceptual and semantic processes; Whatmough et al., 2002; Mion et al., 2010; Ding et al., 2016), bilateral ITG (conceptual lexical retrieval; Indefrey, 2011), bilateral hippocampus (semantic memory retrieval; Sawrie et al., 2000; Binder and Desai, 2011), left IFG (lexico-semantic selection and phonological processes; Binder et al., 2009; Indefrey, 2011) and left pre-central gyrus (syllabification and articulatory processes, output phonology; Indefrey, 2011). Based on naming results, older participants were as accurate as younger ones, suggesting unimpaired sized vocabulary and lexical knowledge. This is consistent with other findings (Villardita et al., 1985; Wierenga et al., 2008; Boudiaf et al., 2016; see Goulet et al., 1994 for a review), allowing us to evaluate possible strategic differences between older and younger adults in manipulating lexical information during object naming. Indeed, a first indication of differential strategies according to age is that, despite normal accuracy, older adults were significantly slower than younger adults. The increased response times in older adults have been frequently reported by other studies and are attributable to various causes, including general slowing of processing speed (Salthouse, 1996; Feyereisen et al., 1998), decline of executive functioning (Craik and Byrd, 1982; West, 2000; Lustig et al., 2007), decline of working memory (Kemper and Sumner, 2001; Waters and Caplan, 2005), decline of perceptual processes (Baltes and Lindenberger, 1997; Schneider and Pichora-Fuller, 2000), and a deficit in lexical access (Bowles, 1989; Barresi et al., 2000; Mirman and Britt, 2013). Lima et al. (1991) proposed that a common general slowing of processing speed can explain aging effects for both lexical (domain-specific) and non-lexical (domain-general) processes. In the same vein, Rogalski et al. (2011) showed that longer RTs for naming in older participants were attributable to a slowing down of general processing, rather than to perceptual or contextual deficits. The idea that naming speed is related to executive and/or other general processes is also consistent with our previous findings (Baciu et al., 2016; Boudiaf et al., 2016) on lexical production with aging. Furthermore, we observed that the OG might be divided into two groups based on naming latencies, the faster OG (OG/RT+) and the slower OG (OG/RT−). The two subgroups differed only on naming latencies, raising the question of what mechanisms led to the situation in which the OG/RT+ group showed shorter RTs than the OG/RT− group in the context of cognitive reserve theory. The following section is divided into two parts, (a) the relation between preserved naming accuracy and HR mechanisms with aging (younger vs. older participants); and (b) the relation between naming latencies and HR mechanisms with aging and between older participants.

As described in the previous sections, our analyses were mainly based on the calculation of two intra- (L_AP_ and R_AP_) and two inter-hemispheric (A_LR_ and P_LR_) HR indices reflecting patterns of reorganization. Based on previous studies, we initially hypothesized that two strategies could be developed by older adults to perform lexical production: a semantic strategy suggesting supplementary involvement of semantic processes (Ansado et al., 2013; Lacombe et al., 2015) and/or an executive strategy assuming that in case of difficulty in performing the task, older adults would recruit executive functions to retrieve and generate words faster (Wierenga et al., 2008). We observed that L_AP_ asymmetry was higher for older participants than younger ones, suggesting the use of a semantic strategy to perform naming with aging. Our findings are in line with Ansado et al. (2013) and Lacombe et al. (2015), as these authors suggest that older participants rely on preserved semantic mechanisms by over-recruiting left temporo-parietal regions to perform the task. We posit that this result, referred as the left anterior-posterior aging effect (LAPA), reflects a domain-specific (linguistic) strategy to maintain performance in older adults for tasks involving semantic processes. However, further studies are needed to determine the direct relationship between semantic processing and the LAPA effect. Our study only shows an indirect relation based on anatomical regions, but has the virtue of highlighting a new HR mechanism that was not discussed otherwise. The LAPA effect was not mediated by naming latencies, reinforcing is compensatory role to naming.

In the older group, participants with faster naming latencies (OG/RT+) showed reduced anterior inter-hemispheric asymmetry as compared to participants with slower latencies (OG/RT−). This suggests a compensatory role for the bilateral frontal regions, in line with the HAROLD model (Cabeza, 2002; Cabeza et al., 2002). Similar effects and interpretations were previously revealed for other processes, such as episodic memory, working memory, and inhibitory control (see Cabeza, 2002 for a review). Altogether, these findings indicate that bilateral frontal recruitment reflects domain-general, unspecific compensatory mechanisms occurring with age. Cappell et al. (2010) reported that the recruitment of right prefrontal cortex for a verbal working memory task was dependent on the task demands in older participants. Specifically, the right prefrontal cortex was additionally recruited for low task-demands and was associated with normal performance. With higher task demands, the recruitment of prefrontal cortex decreased in parallel with performance, reflecting its failure to adapt to increased task demands above a certain threshold. In our study, the over-activation of right frontal regions in faster older adults may reflect compensatory recruitment of executive and attentional processes (Weissman and Banich, 2000). However, when task demands become too high, older participants fail to recruit the right hemisphere, resulting in increased naming latencies. In relation to the cognitive reserve theory (Stern, 2002, 2009), we suggest that the ability to recruit bilateral frontal regions during naming reflects higher neural networks flexibility to cope with higher task demands. These preliminary results need to be validated by further experiments to understand better the differential recruitment of frontal executive functions in older adults, according to their performance and the level of task-demands. Indeed, task demands are classically induced by the task (e.g., low demand task conditions vs. high demand task conditions). For example, it would be interesting to evaluate the effect of task-demands induced by the task (e.g., using low and high frequency words) to determine the relationship between bilateral frontal recruitment (HAROLD model) and the ability to cope with task-demands (cognitive reserve).

Overall, our results suggest at least two co-existent HR patterns and two strategic mechanisms at play in object naming, according to age and to performance: the LAPA effect and the HAROLD model. The newly described model, the LAPA effect, entails an intra-hemispheric left asymmetry with greater involvement of temporo-parietal regions explained by supplementary access to semantic resources engaged to perform a lexical production task. In contrast, the HAROLD model, also tested in older adults, is related to more domain-general mechanisms, consisting of supplementary recruitment of frontal executive regions to maintain a good level of performance. Our findings—mainly the LAPA effect—add to other existing models already described in the literature, such as the CRUNCH (Reuter-Lorenz and Cappell, 2008) and STAC (Reuter-Lorenz and Park, 2014 for a revised version) models. However, these models suggest that multidimensional sources may contribute to the diverse mechanisms and strategies developed with aging, such as anatomo-functional properties (e.g., brain volume, white matter integrity, neural specificity), functional reorganization (e.g., bilateral frontal recruitment, neurogenesis), life experience (e.g., intellectual activities, stress), and learning or training. Thus, assessment of aging effects on cognitive tasks, should be considered in the context of many other factors including occupation, daily and social activities, nutrition, and exercise (Fratiglioni et al., 2004; Paillard-Borg et al., 2009; Allès et al., 2012; Small et al., 2012).

A main limitation of this study is related to the sample size, as only 27 participants took part in our experiment, limiting the statistical power of our analyses. As a consequence, we used non-parametric analyses that are adapted for small sample size, allowing us to compare subgroups (YG vs. OG; OG/RT+ vs. OG/RT−). Furthermore, the small sample size limited our analysis approach to selecting subject-specific ROIs for the computation of HR indices. Hence, having only one group of participants, it was not possible to select activity peaks from one sample, and to analyze BOLD-contrast signal changes from the another. The later approach is more conservative in removing serial testing effects (Kriegeskorte et al., 2010), especially when studying age effects. By using the leave-one-out methodology, we posit that the deep circularity issue, although not totally removed, is diminished (Esterman et al., 2010). Further studies are needed to improve detection sensitivity by increasing the number of participants.

## Conclusion

Our findings suggest that aging has separate and differential effects on the mechanisms and cerebral substrates of lexical production: (a) a LAPA effect with asymmetric anterior-posterior activation and supplementary recruitment of temporo-parietal regions in older adults, suggesting the use of a semantic strategy; (b) bilateral frontal activation (i.e., the HAROLD pattern) in faster older adults, suggesting executive-based and domain-general strategies. The originality of our findings lies in both the description of a new pattern of HR occurring in aging, the LAPA effect, and in underscoring the fact that several mechanisms, involving different strategies and patterns of reorganization might coexist when performing a cognitive task, depending on difficulty, performance and age.

## Author Contributions

EH was involved in design, data analysis, data interpretation, drafting and final approval of the submitted version and approval for all aspects of the project. NB was involved in design and data analysis, final approval of the submitted version and approval for all aspects of the work. EC was involved in data analysis, data interpretation, final approval of the submitted version and approval for all aspects of the project. CP was involved in data analysis, final approval of the submitted version and approval for all aspects of the work. NF was involved in design and neuropsychological evaluation, final approval of the submitted version and approval for all aspects of the work. AK was involved in drafting and final approval of the submitted version and approval for all aspects of the project. AJ was involved in data analysis, data interpretation, drafting work, final approval of the submitted version and approval for all aspects of the project. MB was involved in design, data interpretation, drafting work, final approval of the submitted version and approval for all aspects of the project.

## Funding

This work has been funded by ARC2 “Qualité de vie and Vieillissement” from Région Rhônes-Alpes in France.

## Conflict of Interest Statement

The authors declare that the research was conducted in the absence of any commercial or financial relationships that could be construed as a potential conflict of interest.

## Footnotes

^1^http://www.fil.ion.ucl.ac.uk/spm/
